# GTP-Cyclohydrolase I deficiency presenting as malignant hyperphenylalaninemia, recurrent hyperthermia and progressive neurological dysfunction in a South Asian child – a case report

**DOI:** 10.1186/s12887-019-1580-x

**Published:** 2019-06-15

**Authors:** Kavinda Chandimal Dayasiri, Nayani Suraweera, Deepal Nawarathne, U. E. Senanayake, B. K. T. P. Dayanath, Eresha Jasinge, Kumudu Weerasekara

**Affiliations:** 1 Lady Ridgeway Hospital for Children, Colombo, Sri Lanka; 2 North Colombo Teaching Hospital, Ragama, Sri Lanka

**Keywords:** GTPCH deficiency, Recurrent hyperthermia, South Asian child

## Abstract

**Background:**

Tetrahydrobiopterin (BH_4_) deficiencies are disorders affecting phenylalanine homeostasis, and catecholamine and serotonin biosynthesis. GTP-Cyclohydrolase I deficiency (OMIM 600225) is an extremely rare variant of inborn error of BH_4_ synthesis which exists in recessive and dominant forms. The recessive form presents with complex neurological and autonomic dysfunction whilst the dominant form presents as Dopa-responsive dystonia.

**Case presentation:**

We describe a South Asian child who initially presented with neurological dysfunction and recurrent vomiting and later developed recurrent hyperthermia for several months. The child did not have screening for hyperphenylalaninemia at birth and was found to have marked hyperphenylalaninemia after clinical presentation at 5 months. Further evaluation revealed BH_4_ deficiency. GTP-Cyclohydrolase I deficiency (GTPCH) was identified based on normal dihydro pteridine reductase activity and markedly reduced neopterin in dried blood spot test. After institution of treatment and control of high phenylalanine levels, clinical deterioration decelerated yet with noticeable residual neurological dysfunction.

**Conclusion:**

To authors’ knowledge, this is first report of GTPCH deficiency in a South Asian child. The case highlights practical issues regarding diagnosis of GTPCH deficiency, especially in countries without broader universal newborn screening programs for early detection of inherited metabolic disorders. Testing for GTPCH deficiency should be considered for patients with unexplained neurological and autonomic symptoms following initial metabolic screen.

## Background

Tetrahydrobiopterin (BH_4_) deficiencies comprises a heterogeneous group of disorders caused by mutations at one of the genes encoding enzymes involved in the biosynthesis(GTP cyclohydrolase I or 6-pyruvoyltetrahydropterinsynthase) or regeneration (pterin-4a-carbinolamine dehydratase or dihydropteridine reductase) of BH_4_ [1]. In addition to hyperphenylalaninemia, BH_4_ deficiency leads to dopamine and serotonin depletion in the central nervous system [2]. Most late-diagnosed and untreated BH_4_-deficient patients develop progressive neurological dysfunction from early infancy [3]. Consequences of delayed diagnosis and treatment are devastating and affected children develop clinical features such as mental retardation, seizures, dystonia, drowsiness, irritability, recurrent hyperthermia without infections, hypersalivation, and swallowing dysfunction [4]. Most of these clinical manifestations occur secondary to deficiencies of dopamine, norepinephrine and serotonin.

GTP-Cyclohydrolase I Deficiency (GTPCH − 1) Disorder, a very rare inherited metabolic disorder, is one of the causes of malignant hyperphenylalaninemia due to tetrahydrobiopterin deficiency. The disorder is caused by mutation(s) in the GCH1 gene that encodes for the enzyme GTP Cyclohydrolase I. Currently there are two forms of GTPCH − 1 Deficiency. The first and more common is the autosomal dominant form, also known as Segawa Disease. This form typically responds well to dopamine replacement therapy. The second form is rare and is an autosomal recessive form which does not respond as well to treatment for currently unknown reasons. In countries which offer universal newborn screening for inherited disorders, patients with these rare metabolic diseases are suspected based on the presence of hyperphenylalaninemia.

We describe a South Asian infant who presented with progressive neurological dysfunction secondary to malignant hyperphenylalaninemia and in whom tetrahydrobiopterin deficiency was confirmed and autosomal recessive GTPCH-1 was identified based on normal dihydro pteridine reductase activity and very low neopterin levels. His sibling, who had similar clinical features, succumbed following severe neurodevelopmental regression.

## Case presentation

Five month old boy born to second degree consanguineous parents was brought for evaluation of global developmental delay since birth, and remittent fever, recurrent seizures and vomiting since three months of age. The pregnancy had been unplanned. Though the antenatal period was uncomplicated child had a low birth weight- 2.2 kg (<− 3SD) and had evidence of symmetrical intrauterine growth retardation (OFC - 33 cm/ <5th centile, length – 46 cm/ (− 2) - (− 3) SD). He had recurrent vomiting since two months of age associated with poor weight gain. The clinical course was complicated with recurrent seizures since three months and the child had developmental regression with poor visual fixation and loss of social smile. He had remittent fever for several months with repeatedly negative septic screen following which a central cause for irregularities in thermoregulation was suspected. Later in the clinical course, he developed marked dystonia and dyskinetic movements suggesting extrapyramidal nervous system involvement. His elder sibling who had developmental regression and epileptic encephalopathy, died at 1 ½ years of age following aspiration pneumonia whilst being evaluated for a neuro-metabolic disorder.

**Fig. 1:**
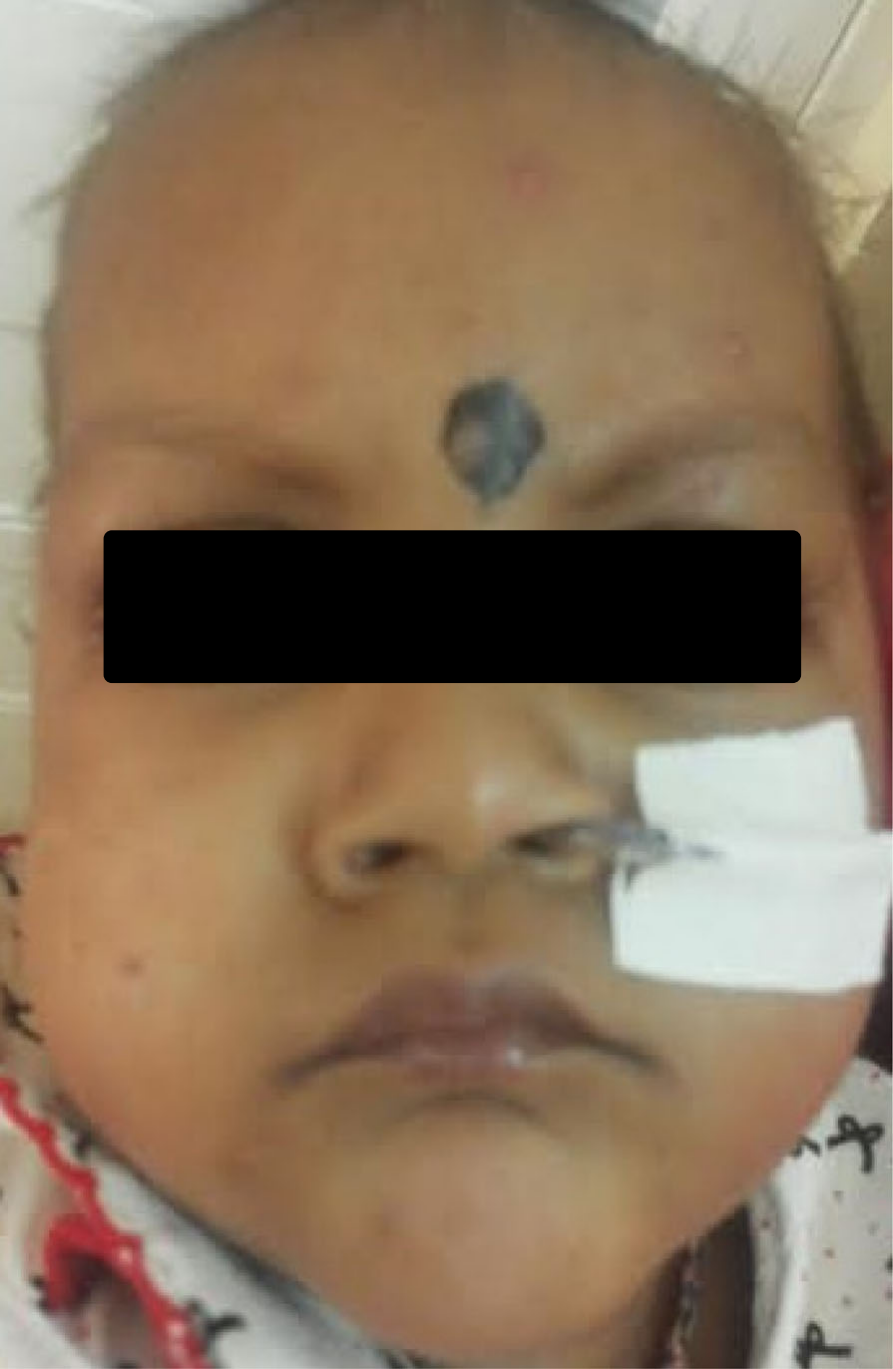
- Absence of phenotypic facial features of phenylketonuria and normal skin pigmentation

**Fig. 2:**
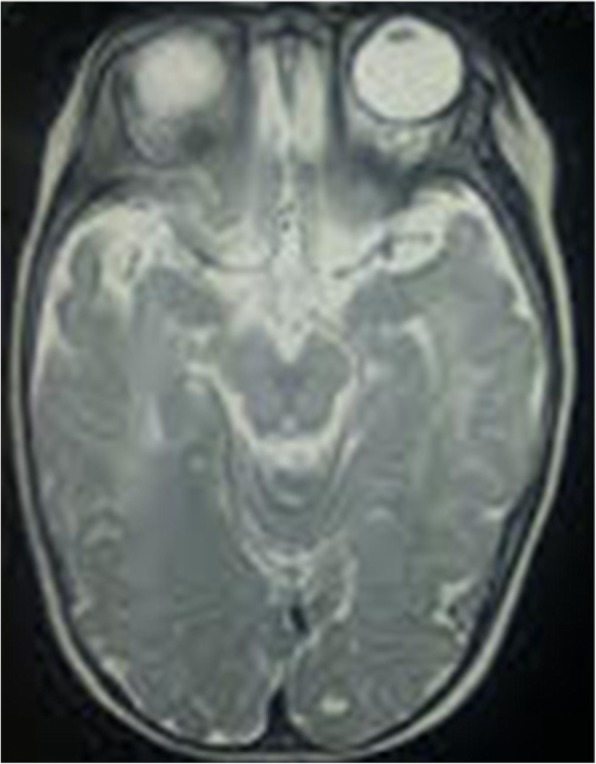
- Segmental demyelination of brain stem, basal ganglia and white matter tracts (upper cross section)

**Fig. 3:**
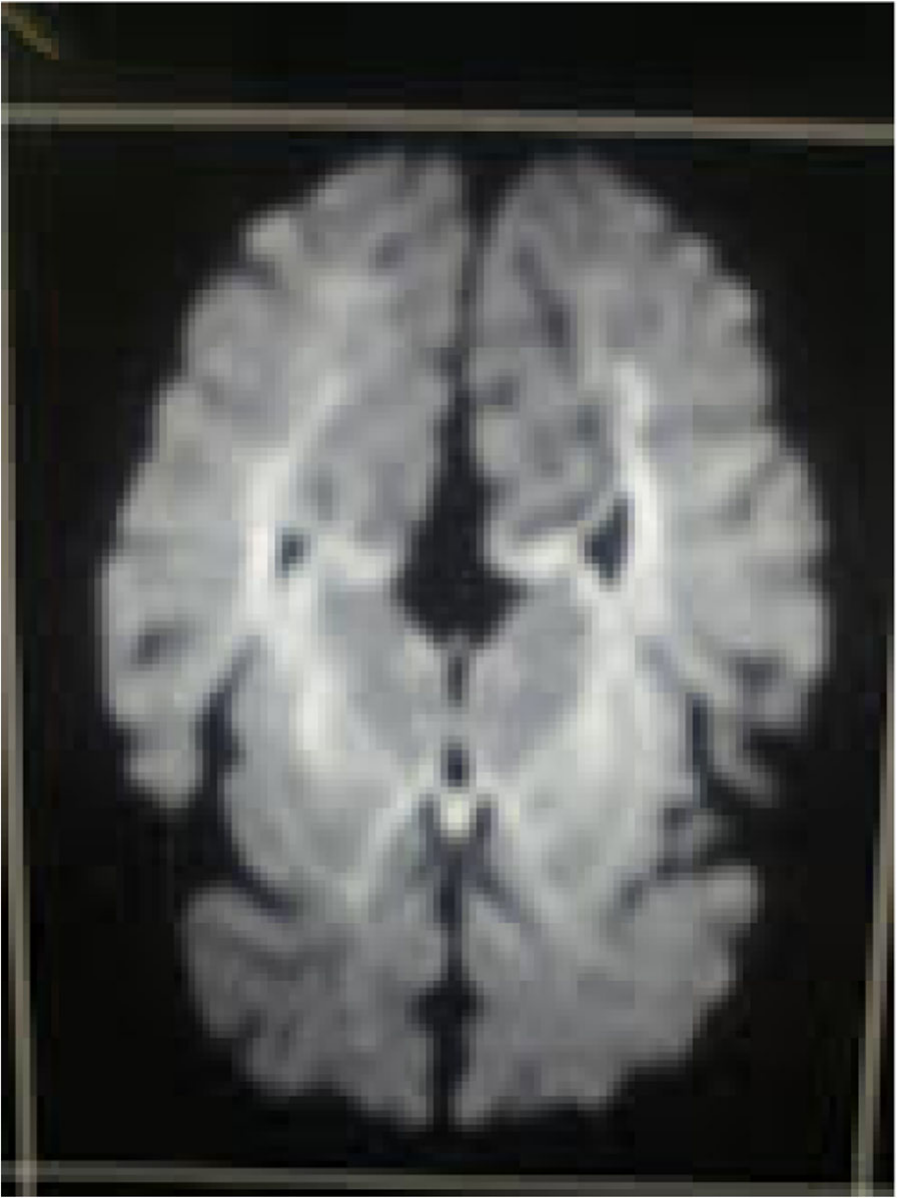
- Segmental demyelination of brain stem, basal ganglia and white matter tracts (lower cross section)

Clinical examination at five months revealed severe growth retardation: weight – 5 kg (<−3SD), length – 59 cm (<−3SD), OFC- 39 cm (<5th centile). He had four limb spasticity and exaggerated reflexes. Electroencephalography revealed encephalopathy. Brain MRI showed multiple areas of increased T2 signal intensity with diffusion restriction involving brain stem, basal ganglia and white matter tracts and suggested widespread demyelination. Urine FeCl_3_ test was positive for phenylketonuria. Plasma amino acid profile revealed elevated Phenylalanine – 1245.71 μM (range 25–120 μM). Plasma Phenylalanine/ Tyrosine ratio was 21.87 (range 0.4–2.2). Blood Neopterin levels were very low – 0.01 nmol/g Hb (range: 0.19–2.93 nmol/g Hb, Haemoglobin – 9.8 g/dl) and Biopterin were not detectable (range: 0.08–1.20 nmol/g Hb, Haemoglobin – 9.8 g/dl). Dihydro Pteridine reductase activity was normal – 2 mU/mg Hb (range - > 1.1 mU/mg Hb). Plasma acylcarnitine profile and screening for other metabolic disorders were negative. Genetic studies were not performed due to lack of facilities and as the biochemical tests were sufficient enough to make the diagnosis of GTPCH 1 deficiency (Figs. 1, 2 and 3).

Child was commenced on Phenylalanine restricted diet with supplementation of other essential amino acids by special formula milk. Micronutrients were also supplemented. Breast feeding was continued and complementary feeds were commenced with a carbohydrate rich and phenylalanine restricted diet. L-Dopa was commenced after detection of BH_4_ deficiency and Tetrahydrobiopterin and 5-hydroxytryptophan were requested as they were not available. Multidisciplinary care was arranged with involvement of paediatrician, nutritionist, metabolic specialist, neurologist and geneticist. Since parents wished for a third child they were offered genetic counseling. Plasma Phenylalanine levels were monitored monthly to assess biochemical improvement. Plasma Phenylalanine level and Phenylalanine/ Tyrosine ratio at 9 months were 18.6 μM and 0.35 respectively.

## Discussion and conclusions

The dried blood spot test analysis in this child revealed markedly reduced neopterin and undetectable biopterin confirming the diagnosis of tetrahydrobiopterin deficiency. Normal dihydro pteridine reductase activity and markedly reduced neopterin identified the defect in GTP Cyclohydrolase 1 pathway by ruling out Dihydro Pteridine reductase deficiency and 6-pyruvoyl-tetrahydropterinsynthase (PTPS) related BH_4_ deficiency. Family history of consanguinity and possibly affected previous sibling clinically suggested the more severe and autosomal recessive GTPCH deficiency rather than the less severe, autosomal dominant GTPCH deficiency (Segawa disease).

The child presented in this report had a sibling who had similar clinical features. Unfortunately, the diagnosis of the elder sibling could not be confirmed due to early demise. Lack of clinical vigilance given the rarity of tetrahydrobiopterin deficiencies in the local setting seemed to be a contributory reason for not establishing the diagnosis. Both children in the current report had early onset similar and severe neurological manifestations suggesting malignant hyperphenylalaninemia and severe neurotransmitter deficiencies in the elder sibling.

Most children with BH_4_ deficiencies have uneventful births but present with early onset development delay [2]. The most striking clinical features (100%) of infants and children with autosomal recessive GTPCH deficiency are muscular hypotonia and movement disorders [2]. Multiple disturbances in neurotransmitter function, in this child lead to neuropsychiatric disturbances, and impaired autonomic regulation (recurrent hyperthermia and vomiting).

Microcephaly which was present in this child is a rare manifestation (less than 4%) [5] reported in all forms of tetrahydrobiopterin deficiencies. He had multiple foci of segmental demyelination involving brain stem, basal ganglia and white matter tracts. Myelination abnormalities are recognized MRI features of hyperphenylalaninemia [6] and tetrahydrobiopterin deficiency [7].The size and number of demyelination changes directly relate with blood Phenylalanine concentration. These changes have been reversed by lowering blood Phenylalanine levels [8].

The use of dried blood spot test is more practical and allows for measurements of pterins, dihydro pteridine reductase activity and amino acids from a single specimen [9]. These tests are essential because they allow for the differentiation of all defects of BH_4_ metabolism that present with hyperphenylalaninemia. Hyperphenylalaninemia (plasma phenylalanine > 120 μmol/L) [10, 11], is considered a cardinal feature of classic autosomal recessive GTPCH deficiency.

Several studies have suggested that diagnosis within the first month of life is essential, and the age at which treatment begins determines the prognosis [12, 13]. Studies from developed countries recommend that selective screening for BH_4_ deficiency is essential in every newborn with even slightly elevated phenylalanine levels [14]. This case highlights practical issues regarding diagnosis of GTPCH deficiency, especially in countries without broader universal newborn screening programs for early detection of inherited metabolic disorders. A positive family history and consanguinity are important risk factors which should guide early referral for metabolic screening as performed in this child. Further, patients having hyperphenylalaninemia with extrapyramidal movement disorders and autonomic dysfunction should be considered at risk for GTPCH deficiency or other treatable disorders of BH_4_ synthesis or recycling and warrant detailed evaluation to prevent devastating neurological complications.

Treatment of BH_4_ deficiencies is two-pronged: the control of hyperphenylalaninemia with oral BH_4_ or a low-Phenylalanine diet and the correction of CNS neurotransmitter homeostasis with neurotransmitter precursors (L-dopa/carbidopa and 5-hydroxytryptophan) [15]. The primary aim of BH_4_ therapy is to restore hepatic phenylalanine hydroxylase activity and normalize blood Phenylalanine levels. Treatment should be initiated as early as possible and continued for the lifetime of the patient. Follow up CSF studies are required to determine neurotransmitter levels that should guide treatment with neurotransmitter precursors.

To our knowledge, this is first report of GTPCH deficiency in a South Asian child. The case highlights practical issues regarding diagnosis of GTPCH deficiency, especially in countries without broader universal newborn screening programs for early detection of inherited metabolic disorders. Authors would like to emphasize the need of neonatal screening for hyperphenylalanemias and research for BH_4_ deficiencies even in patients with slight hyperphenylalaninemia. Testing for GTPCH deficiency should be considered for patients with hyperphenylalaninemia who present unexplained neurological and autonomic symptoms.

## Data Availability

The datasets used and/or analysed during the current study are available from KD on reasonable request. However, they will not be shared publicly due to patient confidentiality of such data.

## Abbreviations

BH_4_: Tetrahydrobiopterin
GTCPH: Guanosine Triphosphate Cyclohydrolase
OFC: occipital frontal circumference
OMIM: Online Mendelian Inheritance in Man
SD: standard deviation
